# Supplementary material for: Competition-Exclusion for Manganese Is Involved in Antifungal Activity of Two Lactic Acid Bacteria Against Various Dairy Spoilage Fungi
Source: Microorganisms. 2025 Nov 6;13(11):2543. doi: 10.3390/microorganisms13112543 (PMC12654002; doi:10.3390/microorganisms13112543)
Supplement: Supplementary file 1 [file microorganisms-13-02543-s001.zip › microorganisms-3905922-supplementary.pdf]

Table S1: Antifungal activity of *L. plantarum* L244 and *L. rhamnosus* CIRM-BIA1759 strains against four fungal targets in model yogurt, supplemented or not with 20 trace elements, measured by antifungal inhibition scoring: ++ slight inhibition and – no inhibition (3 replicates / condition).

Supplemented Oligoelement	Concentration (mg/L)	*P. biforme*		*M. racemosus*		*G. geotrichum*		*Y. lipolytica*	
		L244	CIRM-BIA 1759	L244	CIRM-BIA 1759	L244	CIRM-BIA 1759	L244	CIRM-BIA 1759
None (control)		++	++	-	-	-	-	++	-
Copper	1.665	++	++	-	-	-	-	++	-
Iron	14.841	++	++	-	-	-	-	++	-
Magnesium	1066.95	++	++	-	-	-	-	++	-
Manganese	3.06	-	-	-	-	-	-	-	-
Zinc	35.253	++	++	-	-	-	-	++	-
Vitamin D	0.0441	++	++	-	-	-	-	++	-
Vitamin E	9.27	++	++	-	-	-	-	++	-
Vitamin C	25.974	++	++	-	-	-	-	++	-
Vitamin B1	3.06	++	++	-	-	-	-	++	-
Vitamin B2	21.339	++	++	-	-	-	-	++	-
Vitamin B3	13.914	++	++	-	-	-	-	++	-
Vitamin B5	32.472	++	++	-	-	-	-	++	-
Vitamin B6	4.725	++	++	-	-	-	-	++	-
Vitamin B9	1.899	++	++	-	-	-	-	++	-
Vitamin B12	0.0216	++	++	-	-	-	-	++	-
Vitamin K1	0.0342	++	++	-	-	-	-	++	-
Nickel	0.46386	++	++	-	-	-	-	++	-
Molybdenum	3.798	++	++	-	-	-	-	++	-
Cobalt	0.02502	++	++	-	-	-	-	++	-
Vitamin B7	0.00774	++	++	-	-	-	-	++	-

Table S2: Mean lag values (in hours) implemented with the QurvE software for a selection of 25 fungal strains tested after one-week culture at 10°C in whey from yogurts produced with either *L. plantarum* L244 or *L. rhamnosus* CIRM-BIA1759 strain and supplemented or not with Mn at 2.9 μg/g in comparison to controls with or without Mn

Fungal strains	Control	Control + Mn	L244	CIRM-BIA1759	L244 + Mn	CIRM-BIA1759 + Mn
C. parapsilosis	83.09 ± 10.92	96.02 ± 6.75	90.84 ± 11.21	75.33 ± 38.99	69.24 ± 10.80	101.09 ± 4.01
M. guilliermondii	74.97 ± 11.77	89.21 ± 11.12	114.14 ± 4.48	116.80 ± 6.90	59.75 ± 6.95	57.37 ± 5.93
C. suis	78.04 ± 12.64	92.96 ± 6.44	101.85 ± 11.73	102.85 ± 10.58	91.48 ± 5.23	101.92 ± 7.18
P. roqueforti	80.38 ± 2.25	78.56 ± 12.67	76.25 ± 1.94	91.61 ± 1.13	75.72 ± 3.77	90.48 ± 2.49
D. hansenii	28.04 ± 4.99	35.19 ± 2.73	43.14 ± 9.88	48.83 ± 1.85	27.60 ± 1.44	23.88 ± 2.79
M. circinelloides	29.41 ± 0.55	27.29 ± 0.05	34.49 ± 3.91	35.46 ± 3.61	22.36 ± 2.58	26.81 ± 3.64
G. geotrichum	24.36 ± 2.34	22.45 ± 1.18	21.79 ± 1.66	26.65 ± 4.23	18.70 ± 1.16	25.96 ± 0.71
M. racemosus	18.81 ± 0.94	18.73 ± 2.03	20.24 ± 1.58	17.99 ± 1.26	16.29 ± 1.15	18.71 ± 1.97
P. fermentans	35.10 ± 5.17	31.81 ± 5.23	17.69 ± 3.91	16.54 ± 1.49	22.83 ± 4.60	22.62 ± 2.43
C. intermedia	99.54 ± 2.31	80.96 ± 8.52	65.36 ± 38.08	88.78 ± 2.58	72.94 ± 3.93	82.88 ± 10.39
K. lactis	95.56 ± 10.67	94.22 ± 2.50	84.46 ± 5.65	89.28 ± 5.40	104.44 ± 14.28	72.02 ± 4.82
S. candida	100.03 ± 6.80	104.08 ± 5.13	115.33 ± 9.75	100.76 ± 8.11	101.92 ± 4.82	106.18 ± 4.19
P. adametzoides	87.07 ± 15.38	102.71 ± 7.36	103.80 ± 11.94	101.81 ± 13.72	103.10 ± 5.08	112.40 ± 8.59
P. antarticum	38.05 ± 2.20	59.45 ± 7.51	72.92 ± 0.06	73.65 ± 0.71	62.57 ± 1.52	61.54 ± 1.57
C. inconspicua	35.65 ± 0.88	40.39 ± 1.72	34.67 ± 2.31	42.54 ± 5.10	43.37 ± 3.08	36.84 ± 4.56
P. bialowiezense	44.20 ± 4.43	40.42 ± 0.27	54.93 ± 4.21	59.10 ± 3.11	38.20 ± 0.58	38.58 ± 2.18
Y. lipolytica	18.15 ± 1.56	24.56 ± 3.27	25.31 ± 1.84	28.30 ± 3.48	21.82 ± 2.82	22.03 ± 0.96
P. biforme	45.40 ± 2.17	36.62 ± 2.05	49.59 ± 1.11	55.11 ± 1.35	47.05 ± 0.59	51.40 ± 2.50
P. charlesii	90.13 ± 6.18	104.63 ± 7.76	100.74 ± 15.60	115.06 ± 14.79	114.78 ± 3.48	124.40 ± 5.44
P. solitum	48.73 ± 8.22	53.97 ± 1.17	47.89 ± 0.02	64.89 ± 27.52	50.26 ± 6.50	57.13 ± 1.08
R. mucilaginosa	57.65 ± 1.92	59.10 ± 3.73	56.19 ± 6.33	51.74 ± 3.20	60.76 ± 3.50	69.28 ± 0.98
T. asahii	177.10 ± 21.00	189.96 ± 18.13	210.52 ± 15.21	267.67 ± 11.11	171.67 ± 19.92	189.46 ± 23.42
P. pinodella	78.57 ± 9.48	67.60 ± 12.49	112.93 ± 40.76	116.59 ± 77.70	76.80 ± 7.68	67.69 ± 9.80
T. elegans	15.81 ± 0.27	16.92 ± 1.11	17.05 ± 0.61	21.98 ± 2.41	17.27 ± 1.77	18.02 ± 2.48
C. sphaerospermum	30.39 ± 4.42	25.41 ± 3.22	31.08 ± 7.50	42.68 ± 5.14	22.87 ± 1.96	27.61 ± 4.78

supplementation (3 replicates / condition).

Table S3: Mean μ values (in hours^{-1}) implemented with the QurvE software for a selection of 25 fungal strains tested after 1-week culture at 10°C in whey from yogurts produced with either *L. plantarum* L244 or *L. rhamnosus* CIRM-BIA1759 strain and supplemented or not with Mn at 2.9 µg/g in comparison to controls with or without Mn supplementation (3 replicates / condition).

Fungal strains	Control	Control + Mn	L244	CIRM-BIA1759	L244 + Mn	CIRM-BIA1759 + Mn
C. parapsilosis	0.014 ± 0.001	0.013 ± 0.001	0.007 ± 0.001	0.007 ± 0.003	0.016 ± 0.001	0.011 ± 0.001
M. guilliermondii	0.019 ± 0.001	0.020 ± 0.001	0.014 ± 0.003	0.013 ± 0.003	0.023 ± 0.001	0.025 ± 0.001
C. suis	0.021 ± 0.004	0.023 ± 0.003	0.006 ± 0.002	0.005 ± 0.004	0.016 ± 0.002	0.018 ± 0.003
P. roqueforti	0.033 ± 0.001	0.033 ± 0.002	0.029 ± 0.002	0.022 ± 0.002	0.028 ± 0.002	0.021 ± 0.003
D. hansenii	0.031 ± 0.002	0.027 ± 0.001	0.007 ± 0.002	0.016 ± 0.001	0.025 ± 0.001	0.027 ± 0.001
M. circinelloides	0.029 ± 0.002	0.028 ± 0.001	0.019 ± 0.004	0.019 ± 0.002	0.019 ± 0.002	0.018 ± 0.007
G. geotrichum	0.040 ± 0.001	0.043 ± 0.001	0.036 ± 0.002	0.037 ± 0.001	0.037 ± 0.001	0.035 ± 0.002
M. racemosus	0.041 ± 0.002	0.039 ± 0.001	0.037 ± 0.003	0.035 ± 0.002	0.036 ± 0.001	0.034 ± 0.003
P. fermentans	0.029 ± 0.000	0.026 ± 0.002	0.030 ± 0.003	0.030 ± 0.001	0.031 ± 0.002	0.031 ± 0.002
C. intermedia	0.017 ± 0.001	0.016 ± 0.001	0.015 ± 0.003	0.014 ± 0.003	0.016 ± 0.003	0.017 ± 0.002
K. lactis	0.016 ± 0.002	0.019 ± 0.001	0.018 ± 0.002	0.019 ± 0.001	0.020 ± 0.002	0.016 ± 0.001
S. candida	0.012 ± 0.002	0.017 ± 0.000	0.006 ± 0.002	0.005 ± 0.001	0.017 ± 0.001	0.017 ± 0.001
P. adametzoides	0.013 ± 0.001	0.013 ± 0.003	0.001 ± 0.000	0.002 ± 0.001	0.013 ± 0.001	0.014 ± 0.008
P. antarticum	0.013 ± 0.001	0.018 ± 0.004	0.008 ± 0.001	0.007 ± 0.001	0.024 ± 0.001	0.022 ± 0.001
C. inconspicua	0.023 ± 0.003	0.021 ± 0.005	0.028 ± 0.000	0.028 ± 0.002	0.026 ± 0.000	0.022 ± 0.005
P. bialowiezense	0.014 ± 0.003	0.025 ± 0.000	0.005 ± 0.001	0.009 ± 0.000	0.021 ± 0.001	0.020 ± 0.002
Y. lipolytica	0.029 ± 0.001	0.027 ± 0.001	0.023 ± 0.002	0.024 ± 0.001	0.027 ± 0.002	0.028 ± 0.002
P. biforme	0.026 ± 0.002	0.024 ± 0.001	0.020 ± 0.001	0.027 ± 0.003	0.034 ± 0.002	0.035 ± 0.001
P. charlesii	0.007 ± 0.005	0.020 ± 0.002	0.003 ± 0.001	0.003 ± 0.001	0.015 ± 0.001	0.014 ± 0.002
P. solitum	0.026 ± 0.005	0.031 ± 0.002	0.011 ± 0.003	0.006 ± 0.002	0.028 ± 0.003	0.031 ± 0.001
R. mucilaginosa	0.034 ± 0.001	0.030 ± 0.001	0.011 ± 0.002	0.006 ± 0.003	0.034 ± 0.002	0.029 ± 0.002
T. asahii	0.020 ± 0.002	0.015 ± 0.002	0.009 ± 0.001	0.009 ± 0.002	0.014 ± 0.001	0.014 ± 0.002
P. pinodella	0.026 ± 0.005	0.028 ± 0.006	0.011 ± 0.002	0.012 ± 0.001	0.025 ± 0.003	0.024 ± 0.001
T. elegans	0.019 ± 0.003	0.019 ± 0.002	0.020 ± 0.001	0.018 ± 0.001	0.021 ± 0.002	0.018 ± 0.006
C. sphaerospermum	0.023 ± 0.003	0.025 ± 0.002	0.014 ± 0.002	0.008 ± 0.005	0.029 ± 0.001	0.026 ± 0.001

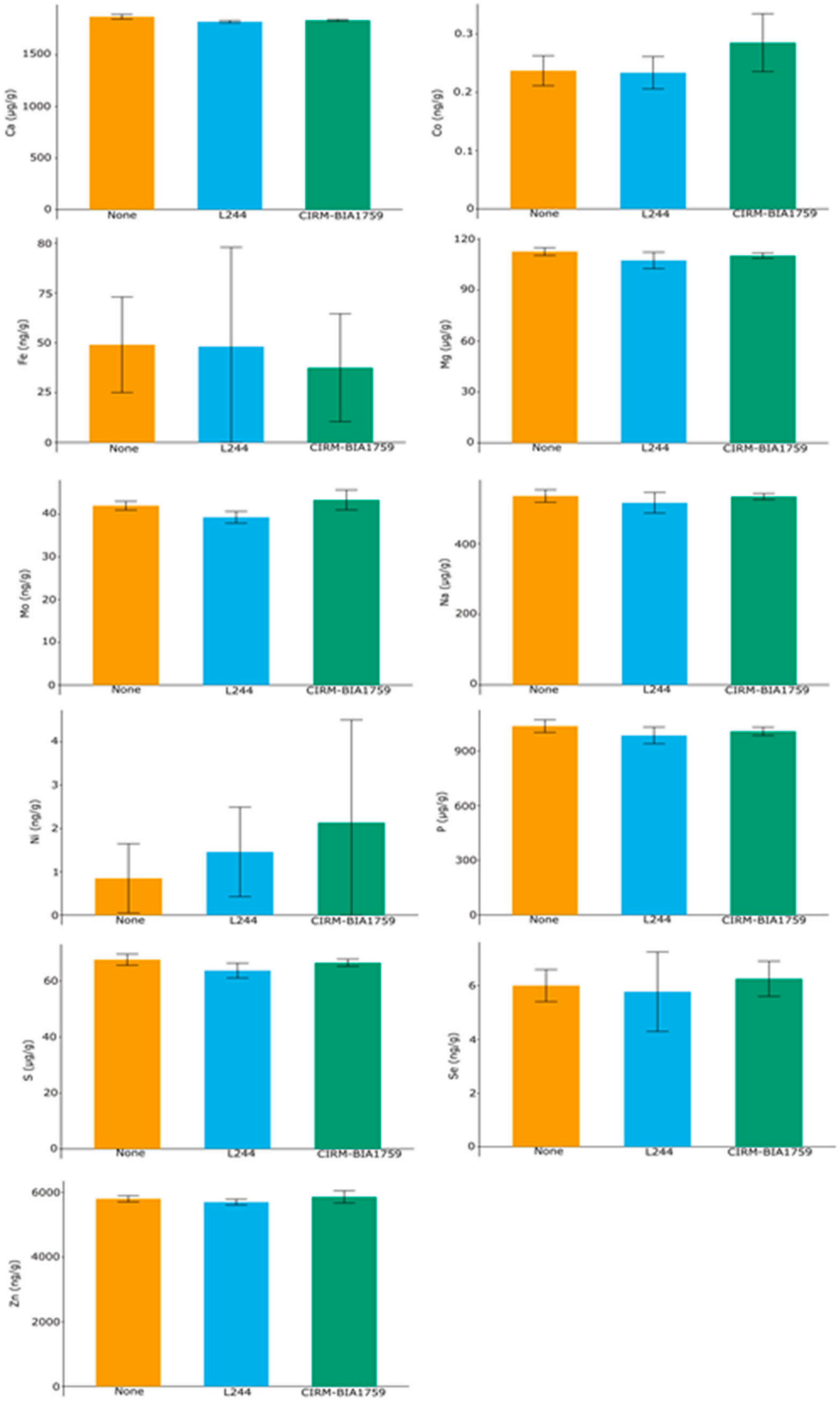

Figure S1: Trace element concentrations (in µg/g or ng/g) determined by HR-ICP-MS in whey from yogurt supplemented with either *L. plantarum* L244 or *L. rhamnosus* CIRM-BIA1759 strains in comparison to the control with only MY800 starters, after two weeks of storage at 10°C (4 replicates / condition).

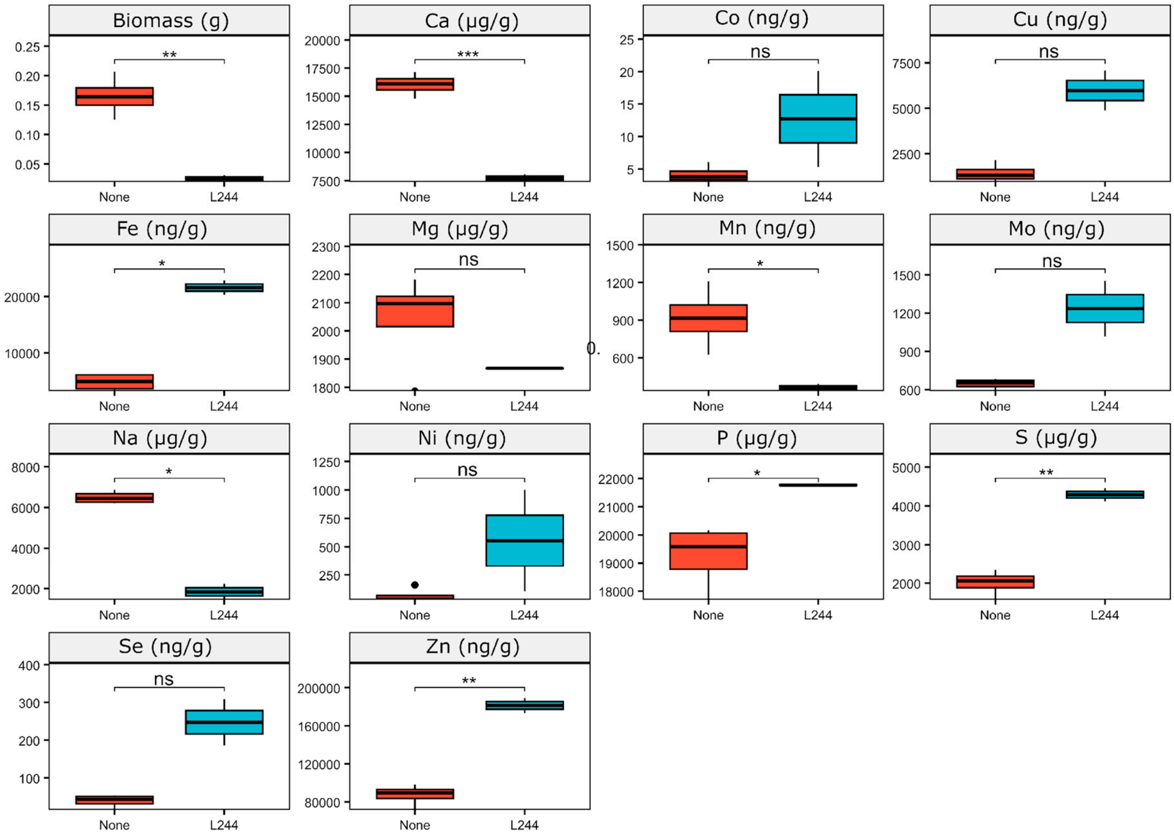

Figure S2: Biomass (in g) and trace element concentrations (in µg/g or ng/g) determined by HR-ICP-MS in *P. biforme* biomass obtained after one-week culture at 10°C in whey from yogurt produced with only MY800 (None), or with addition of either the antifungal *L. plantarum* L244 (L244) or *L. rhamnosus* CIRM-BIA1759 (CIRM-BIA1759) strains (4 replicates / condition).

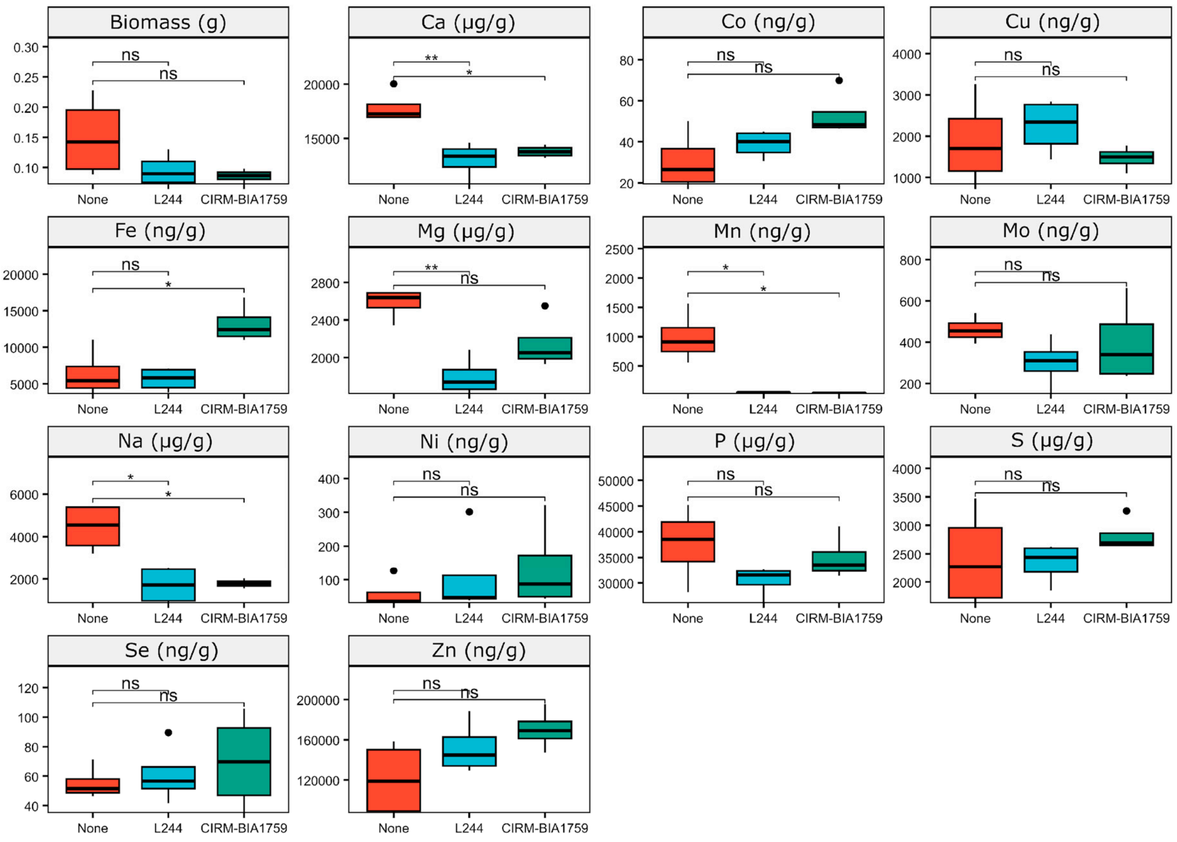

Figure S3: Biomass (in g) and trace element concentrations (in µg/g or ng/g) determined by HR-ICP-MS in *M. racemosus* biomass obtained after one-week culture at 10°C in whey from yogurt produced with only MY800 (None), or with addition of either the antifungal *L. plantarum* L244 (L244) or *L. rhamnosus* CIRM-BIA1759 (CIRM-BIA1759) strains (4 replicates / condition).

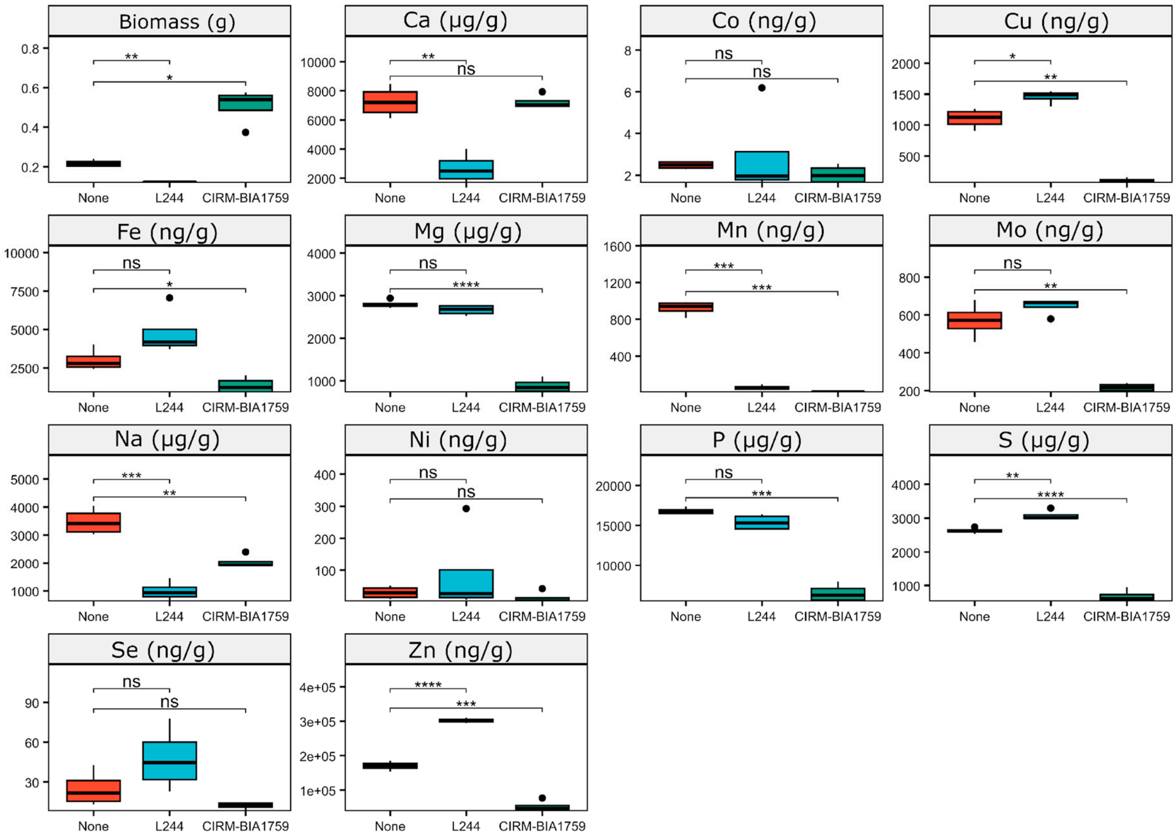

Figure S4: Biomass (in g) and trace element concentrations (in µg/g or ng/g) determined by HR-ICP-MS in *G. geotrichum* biomass obtained after one-week culture at 10°C in whey from yogurt produced with only MY800 (None), or with addition of either the antifungal *L. plantarum* L244 (L244) or *L. rhamnosus* CIRM-BIA1759 (CIRM-BIA1759) strains (4 replicates / condition).

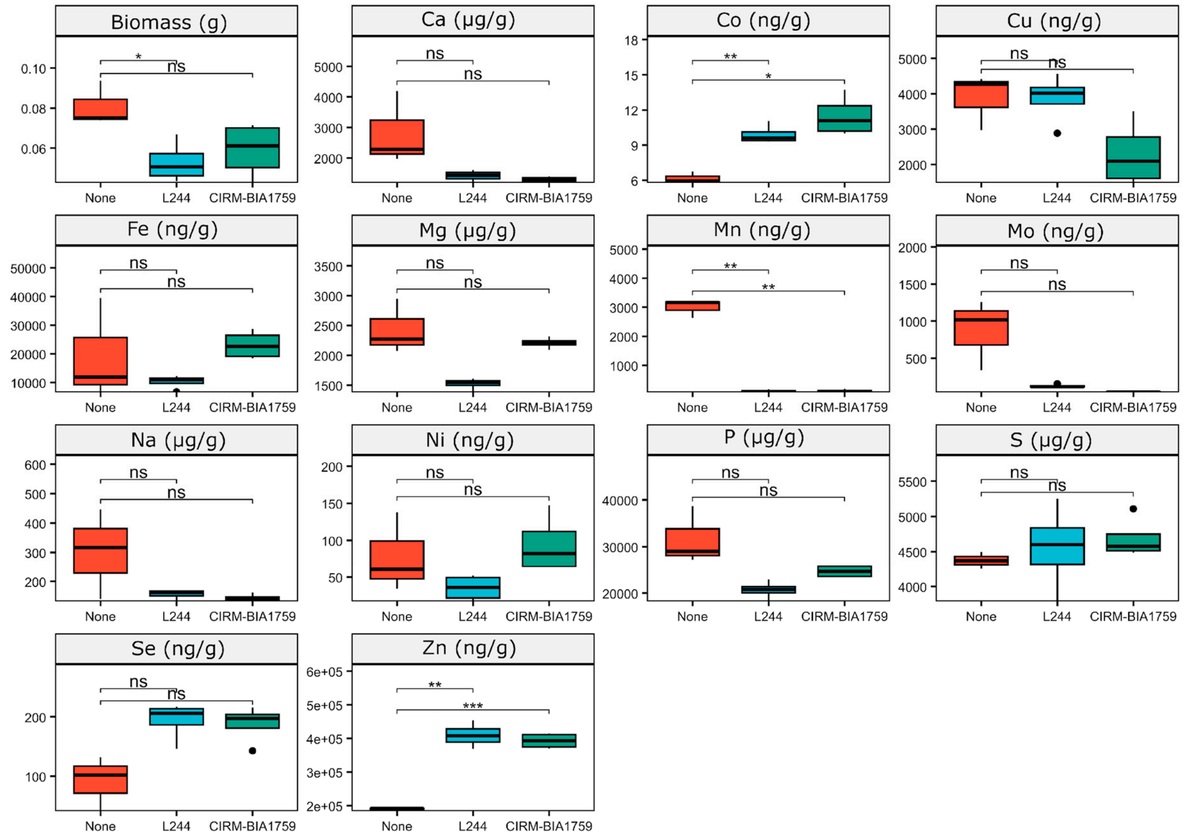

Figure S5: Biomass (in g) and trace element concentrations (in µg/g or ng/g) determined by HR-ICP-MS in *Y. lipolytica* biomass obtained after one-week culture at 10°C in whey from yogurt produced with only MY800 (None), or with addition of either the antifungal *L. plantarum* L244 (L244) or *L. rhamnosus* CIRM-BIA1759 (CIRM-BIA1759) strains (4 replicates / condition).
